# *HOXB9* Overexpression Promotes Colorectal Cancer Progression and Is Associated with Worse Survival in Liver Resection Patients for Colorectal Liver Metastases

**DOI:** 10.3390/ijms23042281

**Published:** 2022-02-18

**Authors:** Eirini Martinou, Carla Moller-Levet, Dimitrios Karamanis, Izhar Bagwan, Angeliki M. Angelidi

**Affiliations:** 1 Department of Hepatobiliary and Pancreatic Surgery, Royal Surrey County Hospital, Guildford GU2 7XX, UK; 2 School of Biosciences and Medicine, Faculty of Health and Medical Sciences, University of Surrey, Guildford GU2 7HX, UK; 3 Department of Bioinformatics, Faculty of Health and Medical Sciences, University of Surrey, Guildford GU2 7HX, UK; c.moller-levet@surrey.ac.uk; 4 Department of Economics, University of Piraeus, 185 34 Piraeus, Greece; karamanis@unipi.gr; 5 Department of Health Informatics, Rutgers School of Health Professions, Newark, NJ 07107, USA; 6 Department of Histopathology, Royal Surrey County Hospital, Guildford GU2 7XX, UK; izhar.bagwan@nhs.net; 7 Department of Medicine, Beth Israel Deaconess Medical Centre, Harvard Medical School, Boston, MA 02215, USA

**Keywords:** HOX, *HOXB9*, colorectal cancer, colorectal liver metastases

## Abstract

As is known, HOXB9 is an important factor affecting disease progression and overall survival (OS) in cancer. However, its role in colorectal cancer (CRC) remains unclear. We aimed to explore the role of HOXB9 in CRC progression and its association with OS in colorectal liver metastases (CRLM). We analysed differential *HOXB9* expression in CRC using the Tissue Cancer Genome Atlas database (TCGA). We modulated *HOXB9* expression in vitro to assess its impact on cell proliferation and epithelial-mesenchymal transition (EMT). Lastly, we explored the association of HOXB9 protein expression with OS, using an institutional patient cohort (*n* = 110) who underwent liver resection for CRLM. Furthermore, *HOXB9* was upregulated in TCGA-CRC (*n* = 644) vs. normal tissue (*n* = 51) and its expression levels were elevated in *KRAS* mutations (*p* < 0.0001). In vitro, HOXB9 overexpression increased cell proliferation (*p* < 0.001) and upregulated the mRNA expression of EMT markers (*VIM*, *CDH2*, *ZEB1*, *ZEB2*, *SNAI1* and *SNAI2*) while downregulated *CDH1*, (*p* < 0.05 for all comparisons). Conversely, *HOXB9* silencing disrupted cell growth (*p* < 0.0001). High HOXB9 expression (HR = 3.82, 95% CI: 1.59–9.2, *p* = 0.003) was independently associated with worse OS in CRLM-HOXB9-expressing patients after liver resection. In conclusion, HOXB9 may be associated with worse OS in CRLM and may promote CRC progression, whereas HOXB9 silencing may inhibit CRC growth.

## 1. Introduction

Colorectal cancer (CRC) is the most common gastrointestinal malignancy and the third leading cause of cancer-related deaths worldwide [1]. Alarming evidence shows that its incidence is rising, especially in the younger population [1]. Despite significant advances in diagnostic and therapeutic strategies, the prognosis remains poor because most patients develop synchronous or metachronous colorectal liver metastases (CRLM) [2]. The development of metastatic disease indicates that cancer cells are not entirely eradicated by current therapies and are the primary cause of cancer-related mortality [2]. CRC is a highly heterogeneous disease which led to the formation of an international consortium in 2015, proposing the molecular classification of CRC into four categories based on transcriptomic characteristics (consensus of molecular subtypes) [3]. In the era of precision medicine, recognising that transcriptomics represents molecular data that are ultimately linked to tumour biology and clinical behaviour, has led to a paradigm shift in the research toward identifying novel transcription factors (TFs) which are linked to the aggressive behaviour of CRC [3]. TFs are important not only in the pathogenesis of CRC but also in the progression and formation of metastases [4]. They also seem to have a prognostic role in overall survival (OS). Thus, they may serve as useful biomarkers and therapeutic targets for the treatment of primary and metastatic CRC [4].

Homeobox containing (HOX) factors are a family of TFs characterised as master regulators of embryonic development that play a pivotal role in regulating cellular functions such as proliferation, invasion, and migration [5]. Humans have 39 HOX genes in their genome, which are organised into four chromosomal clusters (A, B, C, and D), and their importance in cancer has been reported in many studies as alterations in their expression have been found to affect cancer progression [6,7]. The *HOXB9* gene belongs to the HOX family and has been identified as a critical TF involved in numerous human solid tumours as its aberrant expression contributes to tumour growth, progression, and metastases [8]. Several studies have reported that HOXB9 overexpression increases the metastatic potential of cancer cells by activating an important process called epithelial-mesenchymal transition (EMT) [8]. In CRC, EMT is characterised by the loss of epithelial markers (E-cadherin) with the subsequent upregulation of mesenchymal markers (N-cadherin and vimentin) which allows cancer cells to obtain invasive and metastatic potential [9]. Additionally, high HOXB9 protein levels have also been reported by many studies to be associated with a poor prognosis in patients with lung, breast, hepatocellular, and pancreatic carcinoma [8]. In CRC, few studies have reported contradictory findings regarding the HOXB9 prognostic role and function in CRC progression [10]. However, no studies have examined colorectal liver metastases (CRLM) [11,12,13]. Therefore, this study aimed to investigate the impact of HOXB9 on CRC progression and its prognostic importance in CRLM.

## 2. Materials and Methods

### 2.1. Gene Expression Bioinformatics Analysis

To investigate the difference in *HOXB9* expression between cancer and normal tissues in CRC, gene expression data from the Cancer Genome Atlas (TCGA) for colon (TCGA-COAD) and rectal adenocarcinoma (TCGA-READ) were downloaded from the Genomic Data Commons Data Portal (https://portal.gdc.cancer.gov/, (accessed on 23 May 2019)). The edgeR Bioconductor package (v. 3.24.3) was used for data pre-processing and differential expression analyses [14]. A negative binomial generalised log-linear model was fitted to the read counts for *HOXB9*, and likelihood ratio tests for tumour vs. normal tissue differences were conducted using the R package edgeR [14,15]. *p*-values were adjusted for multiple comparisons using the Benjamini-Hochberg (BH) approach [16]. The UALCAN online platform (http://ualcan.path.uab.edu/, (accessed on 23 December 2021) was used to compare the transcriptional levels of *HOXB9* in CRC compared to other types of cancers and the GEPIA tool (http://gepia.cancer-pku.cn/index.html, (accessed on 27 December 2021)) was used to compare the transcriptional levels of *HOXB9* in CRC in comparison with the rest of the *HOX* genes [17,18,19]. Lastly, the OmicSoft Suite with the integrated OncoLand database (Qiagen, Manchester, UK) was used to assess the *HOXB9* gene expression levels in CRC mutant versus wild-type for the top three somatic mutations in CRC which were identified using the COSMIC database (https://cancer.sanger.ac.uk/cosmic, (accessed on 22 June 2020)) [20].

### 2.2. Gene Expression Editing Mechanistic Studies

**Figure 1:**
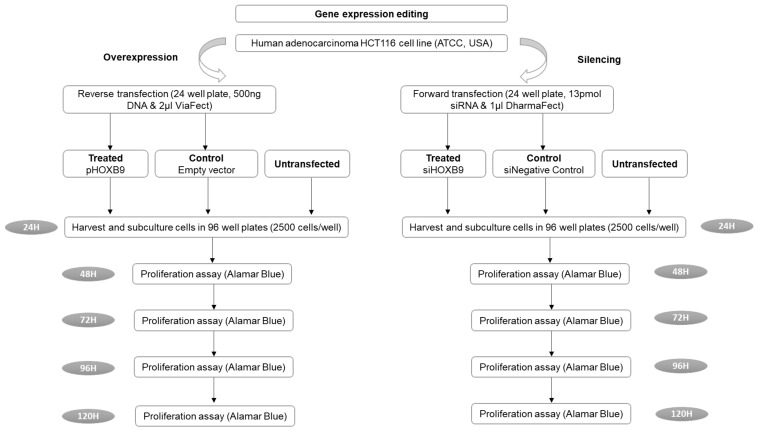
Flow chart of the in vitro experimental studies. (ATCC: American Type Culture Collection, H: hours).

We initially used the STRING server (https://string-db.org/, (accessed on 23 December 2021)) to define HOXB9 functional partners and further explore its potential action. For gene-expression-editing studies, the human HCT116 colon adenocarcinoma cell line was obtained from the American Type Culture Collection (ATCC, Manassas, VA, USA) [21]. The plasmid vectors (OriGene, Köln, Germany), pCMV6-AC-HOXB9-GFP (RG213735), and pCMV6-AC-GFP (PS100010) were used as *HOXB9*, thereby overexpressing negative control vectors, respectively. For *HOXB9* gene silencing, the Silencer^®^ Select small interfering RNA (siRNA) (Life Technologies, Loughborough, UK) against *HOXB9* was used. The non-targeting Silencer^®^ Select siRNA#1 was used as a negative control. The overexpression and knockdown of HOXB9 efficiency were evaluated at the mRNA and protein levels using RT-qPCR and Western blotting, respectively. The outcomes were cell proliferation and fold-change in the RNA expression of EMT markers between the HOXB9 overexpressing cell group and the control group (*VIM, CDH1, CDH2, ZEB1, ZEB2, SNAI1, SNAI2, TWIST*) [22]. Cell proliferation was assessed using the Alamar Blue proliferation assay for up to 120 h post-transfection [22,23]. A detailed methodology of the in vitro studies is provided in Supplementary Digital Content 1.doc (Supplementary Material S1) and the flow chart is shown in Figure 1. The normality of the data was evaluated using the Shapiro–Wilk test. Unpaired two-tailed Student’s *t*-test (for normally distributed values) or Mann–Whitney test (for non-normally distributed values) was used to compare differences between control and treated groups using GraphPad Prism 8 and SPSS v27.

### 2.3. In Silico Transcriptional Regulation Prediction of HOXB9 and Gene Set Enrichment Analysis

We used the Cistrome Data Browser (http://dbtoolkit.cistrome.org/, (accessed on 20 December 2021)) to identify *HOXB9* putative regulators to further dissect its action with regard to CRC proliferation. A gene set enrichment analysis (GSEA) of the gene list identified through the Cistrome DB was performed on the Enrichr server (https://maayanlab.cloud/Enrichr/, (accessed on 2 December 2021)) to identify potentially related biological processes.

### 2.4. Patient Tissue Samples, Clinicopathological Variables and Immunohistochemistry

Approval from the National Research Ethics Committee (Brighton and Sussex REC, Southcoast, 09/H1103/50/AM05) was obtained for the retrospective use of archived formalin-fixed paraffin-embedded (FFPE) human tissue. Available FFPE specimens from patients (*n* = 211) who underwent liver resection for CRLM between 2007 and 2014 were obtained from the institutional archive-management service (http://www.cellnass.com, (accessed on 15 February 2019)). Demographic, clinicopathological, and treatment-related variables were collected from institutional electronic records. Survival data were obtained using the NHS Summary Care Record (SCR) electronic system (NHS Digital, https://digital.nhs.uk/spine, (accessed on 28 October 2020)). Clinicopathological variables were defined based on the Tumour-Node-Metastases staging system (Table 1) [24,25].

Eligible FFPE blocks (*n* = 110) containing viable tumours >40% of the surrounding tissue were selected as donor blocks for tissue microarray (TMA) construction. TMA blocks consisted of 1.5 mm core biopsies taken from the donor blocks and contained CRLM and normal liver tissue [26]. Immunohistochemical staining of TMA slides for HOXB9 was performed with the BenchMark automated Ventana system (Roche Tissue Diagnostics, Dundee, UK), supplementary digital content 1.doc (Supplementary Material S1). Furthermore, HOXB9 expression was semi-quantified by a consultant pathologist blinded to the clinical data, in duplicate, with a cooling period of 4 weeks [27]. Staining intensity was graded as follows, 0: no staining; 1+, weak; 2+, intermediate/strong. The percentage of stained cells was also estimated and the H-score was calculated by multiplying the staining intensity by the percentage of stained cells [28]. To analyse the association of HOXB9 expression with OS in the TMA-CRLM patient cohort, the Reporting Recommendations for Tumour Marker Prognostic Studies (REMARK) were followed and compliance is reported in supplementary digital content 2.doc [27] (Supplementary Material S2).

Sample-size calculation requiring a minimum sample of 43 patients was performed based on previous studies with 85% power and a *p*-value of 0.05 [11,12,29]. Patients were categorised based on their H-Score by selecting the median value of the observed H-Score as a threshold to characterise tumours as H-negative (<10) or H-positive (≥10) [15]. Additionally, among the H-positive patient group, the 30th percentile of the observed H-score range was used to categorise tumours with high expression (≥50) or low expression (<50) [27]. Patients corresponding to core biopsies that were lost during TMA slide cutting (*n* = 11) as well as patients with 90-day postoperative mortality (*n* = 3) were excluded from the final survival analysis. The Kaplan–Meier curves were produced and log-rank test was conducted to compare OS between different groups based on their HOXB9 expression (intensity, cell percentage and H-score). Univariable Cox regression was performed to identify variables that were associated with OS. Multivariable Cox regression analysis was conducted to adjust for competing prognostic factors. Various multivariable models were built containing HOXB9 expression as well as statistically and/or clinically significant variables, which were identified from the univariable analysis [27,30]. Each multivariable model was assessed for “goodness of fit”, with the Omnibus test of model coefficients producing the model’s p-value. Models with a *p*-value ≤ 0.001 have been reported [31]. Analysis was performed using the SPSS package v27.

The association between HOXB9 expression and clinicopathological characteristics was also explored. Three groups were compared based on their HOXB9 expression: (1) negative: H-score < 10, low: H-score (10–50) and high: H-score ≥ 50. Differences in continuous variables were compared using one-way ANOVA, whereas in categorical variables with 2 × 3 Fisher’s exact test using GraphPad Prism 8.

## 3. Results

### 3.1. HOXB9 Differential Expression in CRC

**Figure 2:**
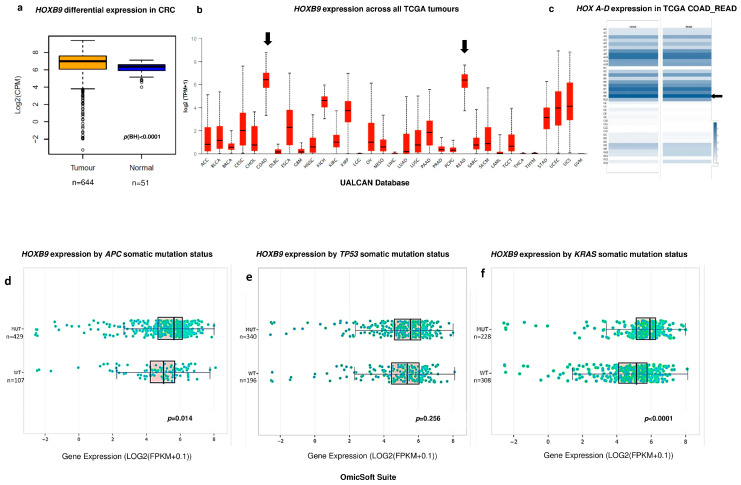
Differential *HOXB9* expression in CRC. (**a**) Box plot of bioinformatics differential HOXB9 expression in CRC TCGA samples vs. normal tissue samples. Values are expressed in Log2 counts per million (Log2CPM). (**b**) Box plot graph produced by the UALCAN web computational server showing the *HOXB9* gene expression levels across all types of cancers in the TCGA datasets. Black arrows represent the expression levels of *HOXB9* in the COAD (colonic adenocarcinoma) and READ (rectal adenocarcinoma) datasets. Values are shown as Log2transcripts per million (log2TPM). (**c**) Expression intensity of 39 *HOX* genes in CRC from COAD (left column) and READ (right column) datasets. Colour intensity corresponds to the value of z-score automatically produced by GEPIA server, black arrow indicates the *HOXB9* gene (**d**) Box plot of *HOXB9* differential expression in *APC* mutant CRC samples vs. wild type CRC. (**e**) Box plot of *HOXB9* differential expression in *TP53* mutant CRC samples vs. wild type CRC. (**f**) Box plot of *HOXB9* differential expression in *KRAS* mutant CRC samples vs. wild type CRC, values expressed as Log2Fragments Per Kilobase of transcript per Million mapped reads (Log2(FPKM + 0.1)). Box plots of figures (**c**–**e**), as well as the *p*-values, were automatically generated by the OmicSoft Suite/OncoLand platform (Qiagen, UK) by selecting the TCGA COADREAD dataset group and the gene-expression command.

There were 644 primary solid tumours and 51 normal samples available in the combined TCGA COAD and READ datasets. Bioinformatics analysis showed that HOXB9 expression was significantly increased in CRC vs. normal colon (*p* < 0.0001), Figure 2a. Additionally the UALCAN platform showed that *HOXB9* demonstrated the highest expression levels in CRC among all types of cancers as shown in Figure 2b whilst the GEPIA tool showed that *HOXB9* demonstrated the highest expression levels in CRC in comparison with the rest of the *HOX* gene family, Figure 2c. The COSMIC database identified *APC*, *TP53*, and *KRAS* as the top three somatic CRC mutations with frequencies of 51%, 46%, and 34%, respectively. The OmicSoft analysis revealed that *HOXB9* expression was higher in mutant CRC versus wild type with highly significant upregulation in *KRAS*-mutated samples (*p* < 0.0001) (Figure 2c–e).

### 3.2. Impact of HOXB9 Dysregulation in CRC Progression In Vitro

**Figure 3:**
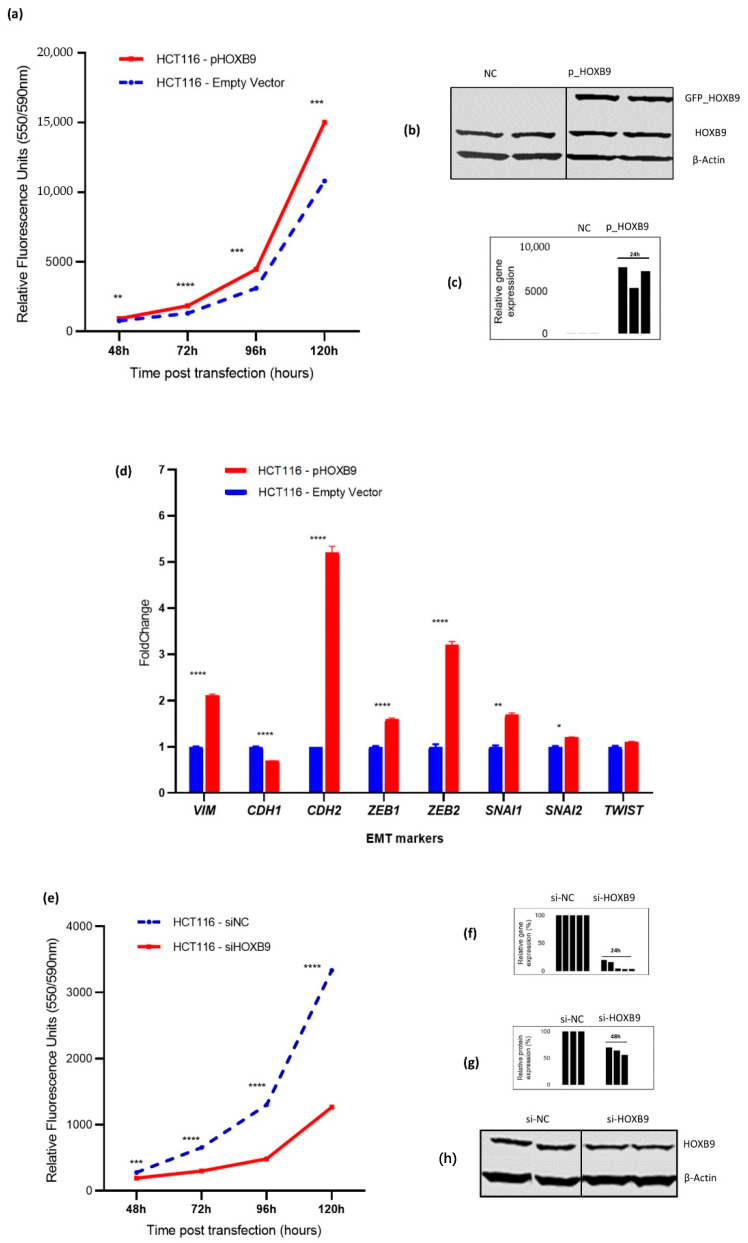
Impact of *HOXB9* gene expression modulation on HCT116 cell proliferation and EMT markers expression in vitro. (**a**) HCT116 cell proliferation measured as relative fluorescence (RFU) after HOXB9 overexpression. Comparison groups were pHOXB9 (overexpressing) and the control group which was transfected with an empty vector. Y-axis represents time points post-transfection, (data derived from 3 biological replicates with 8–12 technical replicates). (**b**) Western blot evaluation of HOXB9 overexpression in HCT116 cells. In the pHOXB9 group (right) the top band shows the GFP_HOXB9 fusion overexpressed protein, the middle band shows the endogenous HOXB9 protein expression and the bottom band shows the expression of β-actin which was used as a loading control. (**c**) Histogram showing relative *HOXB9* gene expression assessed by RT-qPCR in HCT116 cells from 3 biological replicates in triplicates (*ACTB* was used as endogenous control gene). (**d**) RNA fold change expression of EMT-related transcription factors in HCT116 overexpressing HOXB9 vs. control group, (data derived from 3 biological replicates assessed in triplicates). (**e**) HCT116 cell proliferation measured as relative fluorescence (RFU) after HOXB9 silencing. Comparison groups were siHOXB9 (silenced) and the negative control group (siNC). Y-axis represents time points post-transfection, (data derived from 5 biological replicates with 8–12 technical replicates). (**f**) Histogram showing the evaluation of *HOXB9* % knockdown at mRNA level 24 h post transfection with RT-qPCR using the ΔΔCq method. Y-axis represents the % of the relative gene expression normalised to si-NC samples, the difference between si-NC and si-HOXB9 columns represents the % knockdown efficiency, (data are derived from 5 biological experiments assessed in triplicates). (**g**) Histogram showing relative % protein expression in the si-HOXB9 samples in relation to si-NC, the difference between si-NC and si-HOXB9 columns represents the % reduction in HOXB9 protein expression 48 h post transfection, β-actin expression was used as a loading control, data derived from 3 biological experiments. (**h**) Western blot evaluation of HOXB9 silencing in HCT116 cells. The top band shows the HOXB9 protein expression intensity, and the bottom band shows the expression of β-actin which was used as a loading control. Values in (**a**,**d**,**e**) are presented as mean ± standard error of mean (SEM) * *p* < 0.05, ** *p* < 0.01, *** *p* < 0.001, **** *p* < 0.0001.

The STRING server showed that the anti-proliferative proteins BTG1 and BTG2 are among the top ten predicted partners that interact with HOXB9. The gain-of-function experiments in HCT116 cells included 194 treated versus 196 control samples. Additionally, HOXB9 overexpression significantly increased cell proliferation in the overexpressing group compared to that in the control group (Figure 3a,b). Additionally, HOXB9 overexpression significantly upregulated the mRNA expression of mesenchymal markers *VIM* (*p* < 0.0001) and *CDH2* (*p* < 0.0001), while downregulating the epithelial marker *CDH1* (*p* < 0.0001). Additionally, the upregulation of important EMT activators *ZEB1* (*p* < 0.0001), *ZEB2* (*p* < 0.0001), *SNAI1* (*p* < 0.01), and *SNAI2* (*p* = 0.018) were also observed (Figure 3c). Loss-of-function siRNA interference experiments consisting of 189 treated versus 179 control HCT116 samples showed that the silencing of HOXB9 markedly suppressed CRC cell proliferation over five days post gene expression modulation (*p* < 0.001) (Figure 3d,e).

### 3.3. Predicted HOXB9 Regulators and Related Biological Processes

Thirteen TFs were found to potentially regulate the transcription of *HOXB9* in CRC (*CDK9, SP1, HEXIM1, CNOT3, TCF7L1/2, TRIM28, TFAP4, MYC, ZBTB17, CDX2*, and *POLR2A*). Enrichment analysis of the predicted HOXB9 regulators with the Enrichr server revealed that biological processes related to the regulation of cell proliferation and cell cycle were among the significantly enriched ones. An interactive illustration of the GSEA results is provided in the link (https://maayanlab.cloud/Enrichr/enrich?dataset=10db55914af0d55c6d4a8ee83c2b3936, (accessed on 27 December 2021)).

### 3.4. Association of HOXB9 with OS in Patients with CRLM

We investigated the clinical significance of HOXB9 dysregulation by exploring the association between HOXB9 protein expression levels and OS in a cohort of patients who underwent liver resection for CRLM. After excluding TMA procedural tissue loss and 90-day mortality, 96 of the initial 110 patients with a mean age of 66 ± 11 years were included in the final survival analysis. Patient demographics, clinicopathological characteristics, and treatment characteristics along with HOXB9 expression are shown in Table 2.

**Figure 4:**
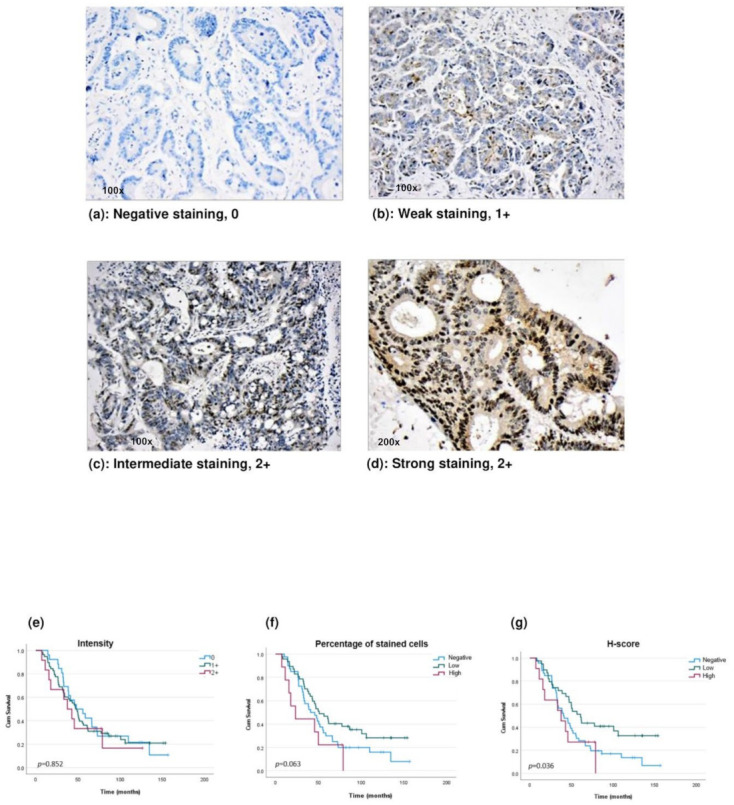
HOXB9 protein expression and OS in patients with CRLM after liver resection. (**a**–**d**) Intensity of HOXB9 protein expression assessed by immunohistochemistry in CRLM tissues. (**e**) Kaplan–Meier curve of OS in CRLM patients based on staining intensity: 0 (blue line), 1+ (green line) and 2+ (red line). (**f**) Kaplan–Meier curve of OS in CRLM patients based on the percentage of stained cells: <10% (negative, blue line), 10–50% (low, green line) and ≥50% (high, red line). (**g**) Kaplan–Meier curve of OS in CRLM patients based on H-score: <10 (negative, blue line), 10–50 (low, green line) and ≥50 (high, red line).

Regarding the HOXB9 protein, no statistical difference was observed in survival Kaplan–Meier curves between patients with a 0, 1+, and 2+ HOXB9 staining intensity (Figure 4a–e). Among patients who expressed HOXB9, those who had a high percentage of stained cells had worse survival compared to patients with a low percentage of stained cells (Figure 4f). Patients were also compared based on their H-scores, as shown in Figure 4g. Among the patients who demonstrated positive endogenous HOXB9 (H-positive) expression, those who had high HOXB9 levels demonstrated significantly worse OS than those in the low-level group (*p* = 0.036). To further evaluate whether HOXB9 expression levels could be a potential independent prognostic factor, a Cox regression analysis was conducted in the patient group who demonstrated endogenous HOXB9 expression (H-positive) (*n* = 50). Univariable Cox regression identified factors that have a prognostic role in OS after liver resection for CRLM and are shown in Table 3. In the univariable analysis, an inverse tendency between HOXB9 levels and OS was found (HR: 2.1; 95% CI: 0.98–4.63, *p* = 0.056) (Table 3). In terms of a multivariable assessment, all three multivariable models showed that among patients who expressed HOXB9, those with high expression seemed to demonstrate an increased risk for worse OS with an HR between 3.8, 95% CI 1.2–12, *p* = 0.023 to 4.2, 95% CI 1.7–10.1, *p* = 0.002 (Table 3). Local recurrence was another factor that demonstrated a significant adverse prognostic role in all three models (*p* = 0.001). Lastly, the size of CRLM ≥5 cm also seemed to increase the likelihood of worse OS (multivariable models 2 and 3) (Table 3).

## 4. Discussion

In this study, we observed that *HOXB9* gene was not only significantly upregulated in cancer vs. normal colon, but its levels were significantly increased when *KRAS* mutations were present. *KRAS* mutant CRC is a molecular subtype of CRC, which demonstrates resistance to standard chemotherapy and immunotherapy [32]. Additionally, KRAS is an established marker of a negative prognosis in patients with primary and metastatic CRC, and the upregulation of *HOXB9* in *KRAS* mutant samples indicates its potential association with aggressive tumour biology [33]. Indeed, studies by Hoshino et al. and Huang et al. have reported a positive association between high HOXB9 protein levels and lymph node invasion, presence of distant metastases, and poor differentiation in patients with CRC [11,12]. Additionally, in our systematic review, we found by conducting a post hoc meta-analysis that high HOXB9 expression levels were associated with a significantly increased risk for metastases (OR 4.14, 95% CI: 1.64–10.43, *p* = 0.003) [10]. In our CRLM patient group, although we did not find a significant association with the adverse CRLM characteristics, we noticed that high HOXB9 levels were positively correlated with the presence of metastatic disease in the regional lymph nodes at the time of the primary cancer resection, indicating that HOXB9 may promote CRC progression and affect survival.

Four studies have demonstrated that HOXB9 significantly affects OS in patients with CRC. Interestingly, studies have shown contradictory results with those of Song et al. [34] and Zhan et al. [13] supporting a favourable prognosis, whereas Hoshino et al. [12] and Huang et al. [11] indicated a negative HOXB9 prognostic role in patients with high HOXB9 levels and with CRC after bowel resection. In our study, we included patients with CRLM after liver resection and our findings are more consistent with studies by Hoshino et al. and Huan et al., as Kaplan–Meier showed that among patients who express HOXB9, those with high staining intensity had worse OS than patients with low levels. Interestingly, we found no difference when patients were categorised based on their staining intensity. The HOXB9 expression level, as an independent risk factor for OS in CRC, has not been previously assessed in multivariable models. Carbone et al. explored the prognostic role of HOXB9 in disease-free survival (DFS) and reported that HOXB9 expression was an independent adverse risk factor for worse DFS in stage IV CRC and possibly more important compared to KRAS and BRAF mutations, which are well-known negative prognostic markers in CRC/CRLM [29,33]. From a bioinformatics analysis that we performed, we also found that patients with high *HOXB9* mRNA levels demonstrated lower DFS survival rates, whereas we observed no difference in OS rates between the high and low *HOXB9* mRNA expressing group (HR: 1 (0.92–1.1), *p* = 0.620, data not shown herein). In our study, in all three multivariable models, a high HOXB9 H-Score and intrahepatic recurrence were the two factors that retained significance as adverse independent prognostic factors in CRLM. The size and number of CRLMs, as well as the development of local recurrence after first liver resection, are well-established prognostic factors in CRLM, indicating that tumour biology plays a vital role in determining prognosis [33,35]. In our study, high HOXB9 levels appear to potentially increase the likelihood of worse OS, similar to the presence of intrahepatic recurrence, which highlights the importance of HOXB9 as a potential prognostic marker in CRLM and suggests that HOXB9 may play an oncopromoting role in CRC. Nevertheless, it has to be acknowledged that to date no definite conclusion can be made regarding the exact association of HOXB9 with OS in patients with CRC indicating the need for further research to elucidate the prognostic role of HOXB9. Additionally, given the fact that stage plays an important role as a selection criterion during a biomarker study, it is suggested that a larger biomarker study restricted to certain stages is needed to further explore the association of HOXB9 with OS in CRC [27].

HOXB9 protein appears to be the most frequently investigated protein among all other HOX proteins in CRC. However, it is interesting that studies report contradictory findings in terms of its clinicopathological significance as well as its mechanistic role in CRC progression. Studies including our own, report opposing findings regarding the association of HOXB9 in OS [10,11,12,13]. This could be attributed to the different methodological approaches implemented by the studies with regard to the categorization of high and low HOXB9 expression patient groups. For instance, despite the fact that studies used IHC as an evaluation method of HOXB9 protein expression, the categorization based on staining intensity varied between studies [10,11,12,13]. Additionally, in our study, we accounted for both the intensity as well as the percentage of stained cells to ensure a more robust classification method of HOXB9 protein expression. Likewise, the experimental observations also differed between studies with regard to the role of HOXB9 in CRC progression. Our study, Huang et al. [11] and Hoshino et al. [12] reported a potential tumour promoting role of HOXB9 whereas Zhan et al. [13] observed a potential tumour suppressive function of HOXB9 in CRC [10]. Variability in the selection of downstream functional assays could be one reason for the contradictory findings. Additionally, HOX proteins undergo significant post-translational modifications which can cause changes in their functions highlighting their potential dual role in cancer [36]. Acetylation has been found by Wan et al. to be an important post-translational modification of HOXB9, resulting in the downregulation of its target gene jumonji domain-containing protein 6 (JMJD6), and subsequently causing a suppression in tumour growth and the migration of in lung adenocarcinoma in vitro [37].

In our gain-of-function experiments, we found that HOXB9 overexpression significantly increased in vitro cell proliferation, indicating a tumour-promoting role; however, the mechanism by which HOXB9 affects cell proliferation in CRC is still unknown. Our protein–protein network analysis showed that important proteins related to cell proliferation may interact with HOXB9. Additionally, TFs that are predicted to regulate the transcription of HOXB9 were enriched in processes related to cell proliferation and the cell cycle, leading to the hypothesis that HOXB9 may play an important role in the cell cycle. This hypothesis is supported by findings from studies conducted in other types of cancer, showing that HOXB9 knockdown results in cell-cycle arrest, indicating that it may be an important molecular component of the cell cycle and may be a promising target for novel personalised gene therapy [38]. Nevertheless, further research in the area of CRC is needed to obtain more evidence on the role of HOXB9 in the cell cycle and cell proliferation. Our study also showed that the RNA expression of important EMT molecular markers and activators was significantly altered. We showed that the mesenchymal markers *VIM* and *CDH2*, which encode for vimentin and N-cadherin, respectively, were significantly upregulated. In contrast, *CDH1*, which encodes the epithelial marker E-cadherin was downregulated. These findings indicate that HOXB9 may contribute to the so-called “cadherin switch”, which is a hallmark of EMT, enabling cancer cells to obtain metastatic potential [39]. Additionally, our experiments showed that the RNA expression of EMT activators such as *ZEB1, ZEB2, SNAI1*, and *SNAI2* was significantly upregulated after HOXB9 overexpression, supporting the hypothesis that HOXB9 may promote CRC progression. Interestingly, HOXB9 has recently been recognised as an important TF that plays a vital role in cancer progression by activating EMT through important signalling pathways, including the transforming growth factor beta (TGF-β) and wingless-related integration site (WNT) signalling pathways [6,7,8]. Furthermore, HOXB9 high expression has been attributed to the promotion of angiogenesis and resistance to anti-angiogenic treatment with bevacizumab in CRC, indicating that silencing HOXB9 could be a promising approach to modulate this resistance [8,29].

To assess whether HOXB9 could be a potential therapeutic target, we transiently silenced its expression, and we observed that the exponential logarithmic growth of HCT116 cells was significantly disrupted in the intervention group. Our in vitro findings are similar to the in vivo findings reported by Hoshino et al. and Huang et al., who also showed that HOXB9 overexpression increased tumour growth, whereas silencing caused the development of fewer lung and liver metastases in nude mice compared to their control group [11,12].

Our study has limitations which should be considered when interpreting its findings. First, this translational prognostic-marker study was based on a small retrospective cohort study. Challenges in optimal biological tissue collection were recognised as FFPE specimens were based on their availability. However, according to our a priori sample calculation based on published studies, our sample size was sufficient to allow for an accurate analysis of our data [11,12]. Additionally, in contrast with the currently published studies, we used various multivariable models to obtain more evidence on the effect of HOXB9 on OS in CRLM, in compliance with the REMARK criteria. Second, in our study, we used the TMA approach to analyze HOXB9 protein expression in CRLM tissues, which potentially introduces selection bias as it consists of core biopsies instead of a larger section and limits the tumour-heterogeneity inspection. In our initial optimization IHC experiments, we noticed that HOXB9 showed heterogeneous staining where some areas were negative, whereas in others, positive staining was observed. Considering this observation, the possible misclassification of a patient as a false negative for HOXB9 expression could not be excluded. Despite the limitations of this approach, TMA is a well-established and widely used technique for biomarker studies and biobanks. To overcome this limitation, we chose the maximum available TMA diameter of 1.5 mm instead of 0.6 mm. Finally, another limitation is that there are no gold-standard classification criteria for immunohistochemical evaluation of HOXB9 expression. To strengthen our study, we used two different categorisation approaches based on staining intensity and H-score. Considering that HOXB9 is emerging as a crucial prognostic factor in various cancers, a consensus to standardise HOXB9 grading in cancers is urgently needed and the above limitations could be potentially minimised by the design of a larger-scale HOXB9 biomarker study. In addition, to validate the hypothesis generated by the survival analysis, we conducted in vitro experiments in addition to our initial bioinformatics analysis.

Our study has several implications which should be explored in future research. CRC/CRLM patients, especially those with KRAS mutations, represent a major treatment challenge and have a worse prognosis [29,33]. Our findings showed that in the HCT116 cell line which harbours KRAS mutation according to the ATCC records, silencing of HOXB9 markedly suppressed cell growth, indicating that HOXB9 may be a novel target for the development of new anticancer agents for resistant CRC/CRLM. The possibility of achieving response and disease control with precision medicine by targeting HOXB9 in a selected group of patients may potentially improve the respectability rates for liver resection and may eventually improve outcomes.

## 5. Conclusions

In conclusion, our study found that HOXB9 may exert an oncopromoting role in CRC by accelerating cell growth and activating EMT. Additionally, our study demonstrates that HOXB9 may play an important role in the OS of patients with CRLM after liver resection. Lastly, we showed that HOXB9 knockdown disrupts CRC cell growth in vitro, indicating that silencing this gene might be a novel approach for the development of personalised gene-directed therapy in primary and metastatic CRC.

## Figures and Tables

**Table 1:** Definition of clinicopathological variables in patients with CRLM.

Variable	Definition
Age (years)	[Date of Operation–Date of Birth]
T	T1–T4, Tumour depth as per American Joint Committee on Cancer (AJCC) 8th edition
N	N0, N1, N2, Lymph nodal invasion as per AJCC 8th edition
M	M0: No metastatic disease at the time of diagnosis of CRC, (liver metastases were developed later: metachronous) M1: Liver metastatic disease present at the time of diagnosis of colorectal cancer (synchronous)
Stage	I–IV, as per AJCC 8th edition
Grade	1: Low differentiation of CRC cells 2: Moderate differentiation of CRC cells 3: High differentiation of CRC cells
Primary Tumour Location	Right site: CRC located from the caecum to the transverse colon up to the splenic flexure Left site: CRC located from the splenic flexure to the rectum
CRLM location	Unilobar: metastases present at either the left or right liver lobe Bilobar: metastases present at both liver lobes
Size of CLRM	Size of largest metastatic deposit measured at histopathological examination (measured in cm)
Number of CRLM	Number of metastatic deposits mentioned at histopathology report
CEA	CEA level measured at the time of the diagnosis of metastatic liver disease (ng/mL)
Response to neoadjuvant chemotherapy	Yes: Patient demonstrating either complete or partial response to chemo on CT according to Response evaluation criteria in solid tumours (RECIST) criteria No: Patient demonstrating either stable disease or disease progression on CT according to RECIST criteria
Resection	R0: resection margin ≥1 mm R1: resection margin <1 mm
Local Recurrence	Patient demonstrating new intrahepatic disease after first liver resection
Overall Survival	Date of death or the date of status checked in the NHS Spine (28 October 2020) minus the date of discharge.

CRLM: colorectal liver metastases, CRC: colorectal cancer, CEA: carcinoembryonic antigen, NHS: National health system.

**Table 2:** TMA CRLM patient cohort demographics, clinicopathological and treatment-related characteristics categorised per HOXB9 expression (H-Score).

	Total (*n* = 96)	Neg: <10 (*n* = 46)	Low: [10–50] (*n* = 39)	High: ≥50 (*n* = 11)	*p*-Value *
Age (mean, SD), [range]	66 (33), [32–81]	68 (11), [32–89]	64 (11), [35–81]	66 (10), [52–82]	0.187
Gender, *n* (%)					
Male	63 (67%)	28 (61%)	25 (64%)	10 (91%)	0.164
Female	33 (33%)	18 (39%)	14 (36%)	1 (9%)	
Deceased	74 (77%)	40 (87%)	25 (64%)	9 (82%)	0.195
	Primary CRC characteristics				
Tumour Location, *n* (%)					
Right colon	15 (16%)	9 (20%)	5 (13%)	1 (9%)	0.402
Left colon	81 (84%)	37 (80%)	34 (87%)	10 (91%)	
Tumour Depth, *n* (%)					
T1/2	18 (19%)	8 (17%)	9 (23%)	1 (9%)	0.546
T3/4	78 (81%)	38 (83%)	30 (77%)	10 (91%)	
Lymph node status, *n* (%)					
Negative	40 (42%)	19 (41%)	20 (51%)	1 (9%)	0.035
Positive	56 (58%)	28 (59%)	18 (49%)	10 (91%)	
Metastases, *n* (%)					
M0	60 (63%)	28 (61%)	23 (59%)	9 (82%)	0.366
M1	36 (37%)	18 (39%)	16 (41%)	2 (18%)	
Stage, *n* (%)					
I/II	17 (18%)	8 (17%)	8 (21%)	1 (9%)	0.680
III/IV	79 (82%)	38 (83%)	31 (79%)	10 (91%)	
Grade, *n* (%)					
Well/Moderate	70 (73%)	36 (84%)	27 (82%)	7 (88%)	0.923
Poor	14 (15%)	7 (16%)	6 (18%)	1 (12%)	
	CRLM characteristics				
CRLM Location, *n* (%)					
Unilobar	65 (68%)	31 (67%)	25 (64%)	9 (82%)	0.537
Bilobar	31 (35%)	15 (33%)	14 (36%)	2 (18%)	
Number of CRLM, *n* (%)					
<4	77 (80%)	37 (80%)	31 (80%)	9 (82%)	0.985
≥4	19 (20%)	9 (20%)	8 (20%)	2 (8%)	
Size of CRLM (cm), *n* (%)					
<5	77 (80%)	37 (80%)	30 (77%)	10 (91%)	0.589
≥5	19 (20%)	9 (20%)	9 (23%)	1 (9%)	
CEA (ng/mL), *n* (%)					
<20	33 (34%)	20 (77%)	12 (100%)	1 (50%)	0.387
≥20	7 (7%)	6 (23%)	0 (0%)	1 (50%)	
Neoadjuvant Chemo, *n* (%)					
Yes	74 (77%)	35 (76%)	30 (77%)	9 (82%)	0.919
No	22 (23%)	11 (24%)	9 (23%)	2 (18%)	
Local Recurrence, *n* (%)					
Yes	31 (32%)	14 (30%)	14 (36%)	3 (27%)	0.865
No	61 (64%)	28 (70%)	25 (44%)	8 (73%)	

**TMA**: tissue microarray, **SD**: standard deviation, **CRLM**: colorectal liver metastases, **CRC**: colorectal cancer, **CEA**: carcinoembryonic antigen, *: 2 × 3 fisher’s exact test.

**Table 3:** Univariable and Multivariable Cox hazards analyses of factors associated with OS after liver resection in CRLM patients who demonstrated endogenous HOXB9 expression (*n* = 50).

Variables	Univariable	Multivariable (1)	Multivariable (2)	Multivariable (3)
	HR (95% CI) *p*-Value	HR (95% CI) *p*-Value	HR (95% CI) *p*-Value	HR (95% CI) *p*-Value
Age	1.02 (0.10–1.04) *p* = 0.121		1.04 (1.00–1.08) *p* = 0.048	1.02 (0.98–1.07) *p* = 0.333
Gender (Male)	1.29 (0.79–2.09) *p* = 0.303			
Local Recurrence *	2.29 (1.40–3.56) *p* = 0.001	4.28 (1.88–9.72) *p* = 0.001	5.73 (2.33–14.08) *p* < 0.001	5.83 (2.11–16.11) *p* = 0.001
HOXB9 staining (2+)	1.18 (0.58–2.43) *p* = 0.648			
HOXB9 H-Score (High)	2.13 (0.98–4.63) *p* = 0.056	3.82 (1.59–9.19) *p* = 0.003	4.15 (1.71–10.06) *p* = 0.002	3.79 (1.20–11.98) *p* = 0.023
Tumour Location * (left)	0.48 (0.26–0.87) *p* = 0.017	0.39 (0.13–1.13) *p* = 0.083	0.38 (0.13–1.10) *p* = 0.074	
Number of CRLM * (≥4)	1.78 (1.03–3.08) *p* = 0.040	1.25 (0.45–3.45) *p* = 0.665	1.41 (0.54–3.71) *p* = 0.489	1.83 (0.58–5.74) *p* = 0.302
Size of CRLM *(≥5 cm)	1.87 (1.08–3.25) *p* = 0.027	2.27 (0.88–5.88) *p* = 0.091	2.76 (1.06–7.20) *p* = 0.038	4.44 (1.11–17.75) *p* = 0.035
T3/4	1.34 (0.64–2.81) *p* = 0.438			
N1/2	1.41 (0.87–2.29) *p* = 0.168			1.04 (0.33–3.28) *p* = 0.946
M1	0.99 (0.51–1.90) *p* = 0.970			
Stage (III/IV)	1.23 (0.64–1.97) *p* = 0.535			
Grade 2/3	1.18 (0.71–1.97) *p* = 0.518			
CRLM Location (bilobar)	1.26 (0.78–2.02) *p* = 0.342			0.42 (0.12–1.46) *p* = 0.170
CEA(≥20 ng/mL)	1.54 (0.79–3.01) *p* = 0.207			
R1 resection	1.09 (0.51–2.35) *p* = 0.827			
Neoadjuvant Chemotherapy	1.26 (0.72–2.23) *p* = 0.422			
Response to Chemotherapy	0.83 (0.42–1.66) *p* = 0.598			

**CRLM**: colorectal liver metastasis, **HR**: hazard ratio, **CI**: confidence interval, T: tumour depth, N: lymph node status, M: metastatic disease, **CEA**: carcinoembryonic antigen.

## Data Availability

Publicly available TCGA COADREAD datasets that were analysed in this study are available through https://portal.gdc.cancer.gov/, (accessed on 23 May 2019). Some datasets generated during the current study are not publicly available but are available from the corresponding author on reasonable request.

## Supplementary Materials

The following supporting information can be downloaded at: https://www.mdpi.com/article/10.3390/ijms23042281/s1.
