# Supplementary material for: HOXB9 Overexpression Promotes Colorectal Cancer Progression and Is Associated with Worse Survival in Liver Resection Patients for Colorectal Liver Metastases
Source: Int J Mol Sci. 2022 Feb 18;23(4):2281. doi: 10.3390/ijms23042281 (PMC8879839; doi:10.3390/ijms23042281)
Supplement: Supplementary file 1 [file ijms-23-02281-s001.zip › Martinou_SDC 1.pdf]

SUPPLEMENTARY MATERIALS AND METHODS

Cell line and Culture

The human HCT116 colon adenocarcinoma cell line was obtained from American Type Culture Collection (ATCC). Cells were maintained in McCoys 5A modified medium (Fisher Scientific, UK), supplemented with 10% fetal bovine serum, 100 µg/ml penicillin and 100 µg/ml streptomycin (Fisher Scientific, Gibco, UK). Cells were cultured at 37°C in a humidified 5% CO2 atmosphere incubator.

Lipid-mediated transient *HOXB9* overexpression *in vitro.*

The overexpression study arm consisted of three groups (**control**: HCT116 cells transfected with the empty vector pCMV6-AC-GFP, **treated**: HCT116 cells transfected with a pCMV6-AC-GFP plasmid containing the cDNA insert of HOXB9, and **untransfected** HCT116 cells).

A transfection mixture was prepared by mixing 500ng plasmid DNA with 2µl ViaFect (Promega, UK) transfection reagent in 100µl OptiMem medium (Life Technologies, UK) and was incubated for 20 mins at room temperature. HCT116 cells were seeded in 24 well plates at $7x10^4$ density containing 100µl transfection mixture to initiate HOXB9 overexpression. After either 24- or 48-hours, cells were harvested, counted and subcultured in 96 well plates at $2.5x10^3$ density for downstream functional assays (AlamarBlue proliferation assay).

siRNA interference *HOXB9* silencing *in vitro.*

The knockdown study arm consisted of three groups (**control**: HCT116 cells transfected with the negative siRNA *Silencer*®Select, **treated**: HCT116 cells transfected with siRNA against HOXB9 *Silencer*®Select and **untransfected** HCT116 cells).

HCT116 cells were seeded in 24 well plates at $5x10^3$ density and were incubated for 24hours. The next day a transfection mixture was prepared by mixing 12pmol siRNA with 1µl DharmaFect (Horizon, UK) transfection reagent in 50µl OptiMem medium (Life Technologies, UK) and was incubated for 20 mins at room temperature. The culture medium was removed from the 24-well plate and was replaced with the transfection mixture in a total volume of 500µl culture antibiotic-free medium. After either 24-hours, cells were harvested, counted and subcultured in 96 well plates at $2.5x10^3$ density for downstream functional assays (AlamarBlue proliferation assay).

RNA isolation and Real-Time Quantitative Polymerase Chain Reaction (RT-qPCR)

Total RNA was extracted from cell line samples using the RNeasy® Plus Micro Kit (Qiagen, UK), according to the manufacturer's instructions. The concentration and purity of RNA were determined spectrophotometrically by measuring its optical density (260/280; 260/230 ratios) using a Nanodrop ND-1000 (Labtech International, UK).

Gene expression was assessed with RT-qPCR using the AgPath-IDTM One-Step RT-PCR Reagents mix (Life Technologies, UK) following the manufacturer's instructions. The reaction was conducted in the Stratagene Mx3005P qPCR machine (Agilent Technologies, USA). TaqMan assays (Life Technologies, UK) containing forward and reverse primers, as well as hydrolysis probes, were selected based on the criteria recommended by thMinimum Ine formation for Publication of Quantitative Real-Time PCR (MIQE) guidelines for *HOXB9* (Hs00256886_m1), *CDH1* (Hs01023894_m1), *CDH2* (Hs00983056_m1), *VIM* (Hs00185584_m1), *SNAI1* (Hs00195591_m1), *SNAI2* (Hs00161904_m1), *ZEB1* (Hs00232783_m1), *ZEB2* (Hs00207691_m1), *TWIST* (Hs00361186_m1), and *ACTB* (Hs01060665_g1). The relative gene expression was calculated using the $2–\Delta\Delta Ct$ method and *ACTB* was used as an endogenous control gene(1). Fold change in gene expression was calculated by dividing the expression level for each gene in each sample with the average gene expression value of the control group.

Western Blotting

Cells were lysed in cell lysis buffer (Sigma, UK) supplemented with protease inhibitor cocktail (Roche, UK) and were quantified using the Pierce™ Rapid Gold BCA Protein Assay Kit (Life Technologies). Equal amounts of whole-cell lysates were separated using the mini-PROTEAN® electrophoresis apparatus (Bio-Rad, UK) at 100V and were transferred on polyvinylidene fluoride (PVDF) membranes. The membranes were blocked with 5% non-fat dry milk for 2 hours and incubated with primary antibodies: anti-rabbit HOXB9 1:1500 (PA5-40576), (Life Technologies, UK) and anti-mouse β-actin 1:2000 (8H10D10), (Cell Signalling Technology, UK), at 4°C overnight. Detection was performed with IRDye 800CW goat anti-rabbit IgG 1:12000 and IRDye 680RD goat anti-mouse IgG 1:12000 (LI-COR Biosciences, UK). Membranes were scanned using Odyssey CLx infrared imaging system (LI-COR Biosciences). Protein bands were quantified using the Image StudioTM Lite Version 4 software (Li-COR, Biotechnology, UK). The band intensity of each protein sample was normalised to the loading control band density (β-actin).

AlamarBlue Proliferation Assay

The Alamar Blue assay is designed to assess the proliferation of various cell lines and incorporates a fluorometric/colourimetric indicator for the detection of metabolic activity of cells. The method is based on the reduction of resazurin (oxidised form) to resofurin (reduced form) by cells' mitochondrial enzymes. Optically, the blue resazurin is transformed into the highly fluorescent red resofurin by viable cells. Fluorescence can be monitored at 530-560nm excitation and 590 emission wavelengths. The fluorescence intensity is proportional to the living cells in the culture.

Cells following gene modulation were subcultured and seeded at desired cell densities in 96 well plates containing 100μL medium per well and were placed for overnight incubation at 37°C at 5%CO2. Cells were inspected for even distribution in the wells with light microscopy. Ten microlitres of AlamarBlue (Life Technologies, UK) were added to each well, including empty wells used as background control. Plates were covered with aluminium foil and were incubated at 37°C. Fluorescent measurements (Relative Fluorescent Units, RFU) were performed on the SpectraMax i3 plate reader (Molecular Devices, UK) at 550 excitation and 590 emission wavelengths. Data were analysed by subtracting the background from the overage fluorescence of each sample.

Immunohistochemistry

Detection of HOXB9 was performed on a 4μm paraffin section with the optimal anti-rabbit HOXB9 (PA5-40576), (Life Technologies, UK) polyclonal antibody dilution (1:150) which was determined on optimisation experiments on human lung tissue as a positive control. IHC staining was performed with the BenchMark automated Ventana IHC system (Roche Tissue Diagnostics, UK) using the pre-diluted dispensers of the *ultra*View Universal DAB (diaminobenzidine tetrahydrochloride) Detection kit (Roche, UK) and included the following steps:

Sections from FFPE blocks were cut using a microtome at 4μm thickness. Subsequently, the slides were baked at 60°C for 30mins for paraffin to melt. For each slide, Ventana labels were printed which included a unique barcode containing all protocol information. The slides were then loaded onto the Ventana IHC system and were further incubated at 72°C for 8mins for further deparaffinisation. Cell conditioning using CC1 solution was performed for a selected time for antigen retrieval. CC1 is a tris-based buffer with a slightly basic pH which at elevated temperatures is capable of disrupting bonds formed by formalin in tissue and allows denaturation of protein molecules and increases antibody accessibility.

Following cell conditioning completion, endogenous peroxidase was inhibited with *ultra*View Universal DAB inhibitor (Ventana, UK) for 4mins. The primary antibody was manually titrated at the desired dilution and was incubated for a selected period. A secondary antibody cocktail was subsequently applied (*ultra*View Universal multimer, Ventana, UK) followed by incubation for 8 mins. Two additional 8mins incubation steps were performed using DAB chromogen and hydrogen peroxide,

which cause an enzymatic reaction producing brown precipitate. Lastly, to enhance the DAB brown colour, copper was applied, followed by 4mins incubation. Each slide was counterstained with haematoxylin for 12mins. Post counterstain, one drop of bluing reagent was applied, followed by an incubation period of 4mins. The slides were then dehydrated and coverslipped using Roche HE600. Staining assessment was performed under light microscopy. The optimal conditions for HOXB9 were 1:150 dilution with antigen retrieval for 52mins, primary antibody incubation for 32mins followed by an amplification step. Each TMA slide contained human lung tissue as a positive control to ensure IHC efficiency.
